# Comparison of risk factors and different therapeutic options for ocular toxoplasmosis recurrence: a retrospective study

**DOI:** 10.1186/s12348-026-00578-x

**Published:** 2026-05-21

**Authors:** Clara Rizzo, Rocco Micciolo, Daniela Bacherini, Fabrizio Giansanti, Erika Bonacci, Giorgio Marchini, Emilio Pedrotti, Francesca Bosello

**Affiliations:** 1 Department of Neurosciences, Psychology, Drug Research, and Child Health, Eye Clinic, University of Florence, AOU Careggi, 50139 Florence, Italy; 2 Centre for Medical Sciences, Department of Psychology and Cognitive Sciences, University of Trento, Trento, Italy; 3 Department of Engineering for Innovation Medicine, Ophthalmology Clinic, University of Verona, Verona, Italy; 4 Department of Surgery, Dentistry, Maternity and Infant, Ophthalmology Clinic, University of Verona, Verona, Italy

**Keywords:** Ocular toxoplasmosis, Disease recurrence, Risk factors of ocular toxoplasmosis

## Abstract

**Background:**

Ocular toxoplasmosis is a leading cause of vision impairment and is burdened by the risk of recurrence. This study, conducted at the University Hospital of Verona, aimed to identify potential risk factors associated with disease recurrence.

**Main Body:**

A total of 86 patients were treated for ocular toxoplasmosis between 1996 and 2023, with 43 completing treatment and follow-up of at least 18 months after treatment. Patients were treated with one of two therapeutic options: either trimethoprim-sulfamethoxazole or pyrimethamine-sulfametopyrazine. Over the study period, 21 patients experienced at least one recurrence, with a median time for the first recurrence of approximately six years. The average follow-up duration was eight years, and the probability of recurrence after seven years was 58%. Sleep duration emerged as a significant risk factor, as patients who slept between six and eight hours per night had a lower likelihood of recurrence. No significant associations were found with other factors, including gender, ethnicity, country of birth, education level, smoking, alcohol consumption, age at diagnosis, autoimmune diseases, vitamin deficiencies, vaccinations, cat ownership, consumption of raw or undercooked meat, place of residence, occupational soil exposure, primary infection (IgM positive), or the affected eye’s laterality. Moderate evidence suggested a potential link between recurrences and psychological factors, such as stressful life events, lesion location, and pregnancy following the first diagnosis. Notably, women who experienced pregnancy after diagnosis had a threefold increased risk of recurrence. Regarding visual outcomes, there was modest evidence indicating that patients treated with trimethoprim-sulfamethoxazole achieved better final visual acuity compared to those treated with pyrimethamine. However, this difference was not statistically significant, and the underlying mechanism remains unclear.

**Conclusion:**

The findings highlight the potential role of sleep duration in reducing recurrence risk and suggest a possible association between psychological stress, post-diagnosis pregnancy, and recurrence. Additionally, trimethoprim-sulfamethoxazole treatment may contribute to better long-term visual acuity, although further research is needed to confirm these observations.

## Background

Ocular toxoplasmosis (OT) is an infection of the eye affecting mainly the retina and choroid caused by the intracellular protozoan *Toxoplasma gondii (T. gondii).* OT is the leading cause of infectious posterior uveitis worldwide [1] and one of the most common causes of vision loss due to intraocular infections. Approximately 25–30% of the global population is systemically infected with Toxoplasma, making it the most common foodborne parasitic infection worldwide [2]. The prevalence of systemic toxoplasmosis varies significantly by region. A low prevalence (10–30%) is observed in North America, Southeast Asia, and Northern Europe, whereas Central and Southern Europe have a moderate prevalence (30–50%) [3]. In Latin America and tropical countries, the prevalence of Toxoplasma infection exceeds 60%, with Brazil identified as one of the countries with the highest rates [2]. Ocular toxoplasmosis typically follows a recurrent course, which can be explained by the parasite’s life cycle. Recurrence rates reported in the literature range from 40% to 79% [4]. The risk of recurrence has been reported as highest in the first year after initial activation and tends to decline in subsequent years [5–7]. Higher recurrence rates have been observed in extreme age groups, including the elderly and very young children [8], as well as in individuals infected with Brazilian *T. gondii* strains compared to European or American strains [9]. Additionally, patients who are IgM-positive and receive intravitreal therapy instead of conventional oral treatment, those lacking long-term antibiotic prophylaxis, and immunocompromised individuals are at an increased risk of recurrence [10]. Other potential risk factors for reactivation include pregnancy, AT heterozygosity in the *IFN-γ* gene at position + 874 [11], and the use of steroids without concurrent antibiotic therapy. Most of these factors can be explained by immune function deterioration [12], which facilitates the reactivation of parasitic cysts localized in the retina. Understanding recurrence rates and risk factors for toxoplasmosis relapse is crucial for guiding prophylactic treatment in high-risk patients. Prophylactic antibiotic treatment for secondary prevention has been shown to reduce recurrence rates. A randomized study comparing long-term prophylactic treatment with trimethoprim-sulfamethoxazole versus placebo over 20 months demonstrated a reduced recurrence rate in treated patients (6%) compared to untreated individuals (23.8%) [13]. As such, individuals with a risk of frequent recurrences of ocular toxoplasmosis may benefit from long-term prophylactic therapy to prevent further relapses. Our study aimed to identify risk factors for the recurrence of ocular toxoplasmosis in a population of patients treated and followed up at the University Hospital of Verona.

## Main text

Our study was conducted as a single-center retrospective analysis of patients treated for unilateral ocular toxoplasmosis activation and subsequently followed for recurrences. The charts of patients treated for OT from 1996 to 2023, living in urban area of Verona or in the surrounding suburbs, were reviewed. A total of 86 patients were treated for OT during this period. Forty-three patients were lost to follow-up within 18 months of therapy cessation and were not reachable by phone, so they were excluded from the study. Forty-three patients completed treatment and had a minimum follow-up of 18 months after treament cessation; they were all immunocompetent and none of them received a long-term prophylactic therapy. 35 of them responded to a questionnaire on risk factors for infection and toxoplasmosis recurrence. Data on patients’ clinical history, characteristics of the acute ocular toxoplasmosis episode (including visual acuity, treatment received, and infection location), as well as recurrences, were collected through a review of medical records. Patients were categorized based on demographic factors (age, sex, ethnicity, country of birth), known primary infection (IgM+), treatments received and time without recurrence after treatment cessation. All patients were treated with either trimethoprim-sulfamethoxazole or pyrimethamine-sulfametopyrazine. Antibiotic therapy was administered in combination with prednisone tablets 50 mg/die tapering. Treatment adherence was assessed, and the therapy was continued for at least 4–6 weeks to ensure the effectiveness of the steroid treatment. The infection site was divided into Zone 1 and Zone 2 according to the classification previously described by Ajamil-Rodanes et al. [14] Zone 1 represents the region with lesions that most threaten vision, encompassing an area extending 2 disc diameters (3600 μm) from the foveal center or 1 disc diameter (1800 μm) from the optic disc margin. Zone 2 extends from the outer boundary of Zone 1 to the ora serrata, outside the vascular arcades. The patients who completed the questionnaire (35 patients) were interviewed regarding various risk factors, including: demographic and clinical factors (education level, main symptom at diagnosis, number of recurrences); comorbidities and immunosuppressive condition (autoimmune disease, immunosuppressive drugs, vitamin deficiencies, vaccinations); behavioral (smoking, alcohol) and environmental factors responsible for possible reinfection (pets, raw meat consumption, residence area at diagnosis, work in contact with soil); psychological factors and stress (sleep hours, significant life changes/stressful events); and only for women, pregnancy-related factors (pregnancy before or after the first ocular toxoplasmosis diagnosis).

The cumulative probability of the first recurrence was estimated employing the Kaplan-Meier product limit estimator [15], while the log-rank test [16] was used to compare recurrence probability among different groups. The Cox model [17] was employed to estimate the hazard ratio of recurrence among different groups; for all these analyses, only the first recurrence was taken into account. Overall recurrence incidence rates were estimated and compared by means of Poisson regression [18]. All the analyses were performed employing the R software (version 4.2.0) [19]. A significance level of 5% was always adopted.

### Results

**Fig. 1:**
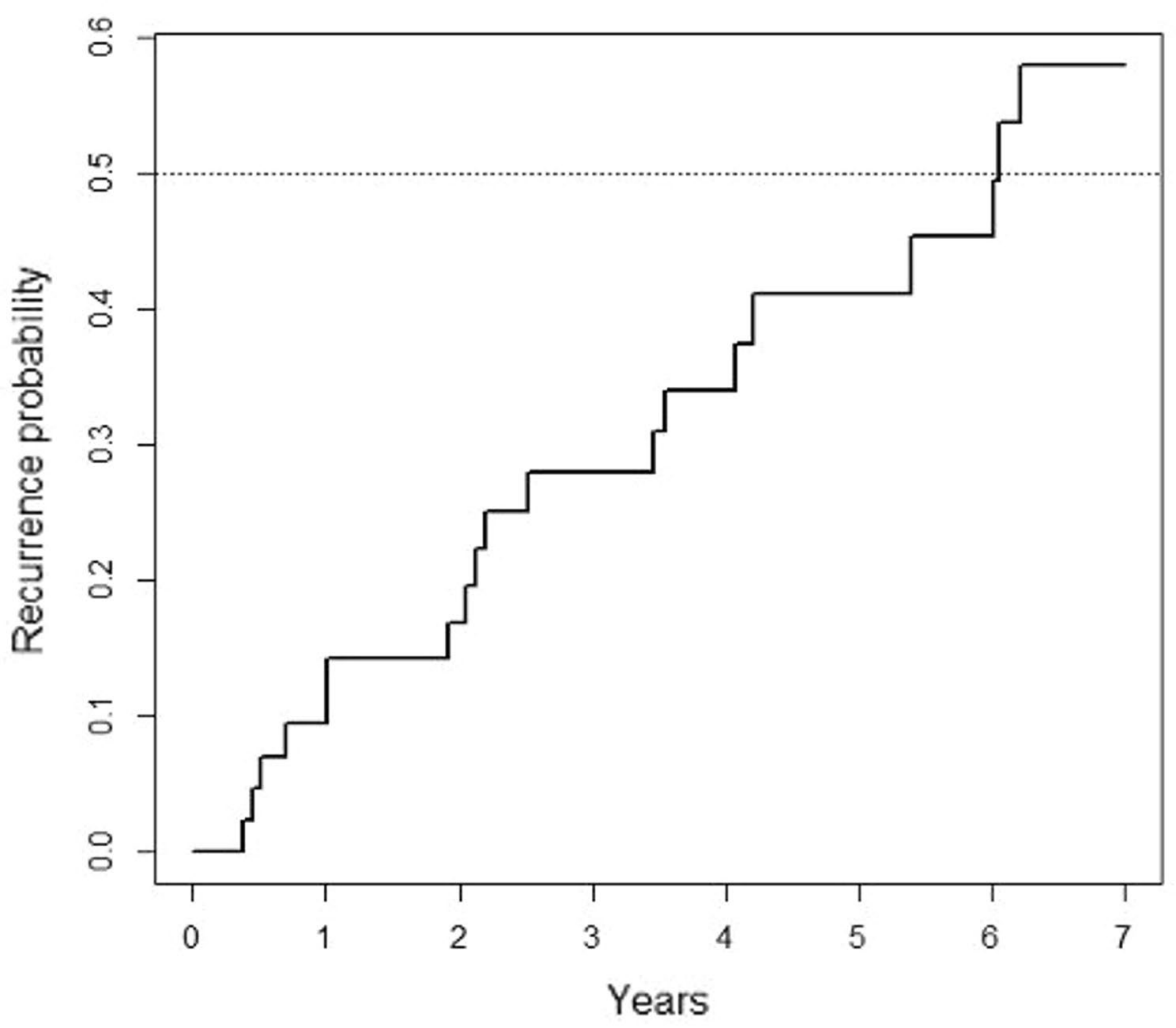
Recurrence probability of ocular toxoplasmosis. The x-axis represents years of follow-up, and the y-axis represents recurrence probability

The median follow-up for patients was 8 years. A total of 21 out of 43 patients experienced at least one recurrence (of these, two subjects had a total of 2 recurrences and 1 a total of 4 recurrences), with the last recurrence recorded almost 11 years after initial diagnosis. Figure 1 shows the Kaplan-Meier estimate of the cumulative probability of the first recurrence over time. The median recurrence time was just over 6 years, indicating that half of the patients had the first recurrence within 6 years of follow-up.

The Kaplan-Meier estimates of the first recurrence probability at different times were as follows (in parenthesis the cumulative number of subjects with the first recurrence are given): 1 year: 9.4% (4 subjects), 2 years: 16.9% (7), 3 years: 27.9% (11), 4 years: 33.9% (13), 5 years: 41.0% (15), 6 years: 45.3% (16), and 7 years: 57.9% (19). At this time, there were still 10 subjects “at risk” (i.e. without the first recurrence); of these, one relapsed after 8 years and one just before 11 years.

Regarding the time interval in which recurrences are most likely to occur, Holland et al. [7] described a high probability of relapse shortly after the first episode, corresponding to a clustering pattern, with a reduction in recurrences at longer time intervals. Similarly, Bosch-Driessen and colleagues [6] reported a cumulative recurrence risk of 29% after one year. However, in our study the first recurrence rate was not higher in the first few years. Within the first 6 years of follow-up, 16 subjects had the first recurrence, while the total follow-up period was about 153 person-years. Therefore, the overall recurrence rate was about 1 recurrence every 10 person-years and, between 0 and 6 years of follow-up, annual recurrence rates were not significantly different each other (Likelihood Ratio Test = 0.83; *p* > 0.5).

No statistically significant differences in the recurrence probability were found when analyzing the following variables: gender, ethnicity, country of birth, education level, smoking, alcohol consumption, age at diagnosis, autoimmune diseases, vitamin deficiencies, vaccinations, pet ownership, consumption of raw or undercooked meat, residence area, work involving contact with soil, IgM positivity at first diagnosis (IgM+), affected eye and medication used (trimethoprim-sulfamethoxazole or pyrimethamine-sulfametopyrazine).

#### Socioeconomic factors

Regarding socioeconomic factors, a low socioeconomic status has been associated with increased exposure risk, reduced access to healthcare services, and delays in acquiring appropriate treatment for infection control. In Brazil, for example, lower educational levels and socioeconomic conditions have been correlated with a higher risk of ocular infection and greater exposure to the parasite [20]. However, in our study, which assessed education level categorized as primary school (elementary), lower secondary school (middle school), upper secondary school (high school), and university education, as the primary socioeconomic indicator within our study population, no higher incidence of ocular toxoplasmosis recurrence was observed in patients with lower socioeconomic status. Despite the potential association between these factors and infection risk in other studies, our findings did not indicate that socioeconomic factors, such as education level had a significant impact on the recurrence rates in this patient population.

#### Gender

Regarding gender, some authors have reported a higher incidence of toxoplasmosis in women [21]. Other studies, such as that by Neto et al. [22], have suggested a higher prevalence in men, with this difference being attributed to greater consumption of raw meat or exposure to oocysts in the soil. However, in our study, no significant gender-related differences in recurrence probability were observed (log-rank test = 0.04; *p* > 0.5).

#### Ethnicity and country of birth

Regarding ethnicity, ocular toxoplasmosis in immunocompetent individuals in South America has been described as more severe compared to other continents [20, 23, 24]. This has been linked to infection with more virulent strains of *T. gondii*. Parasitic strains from South America are genetically more diverse and contain virulent alleles more frequently than strains from other continents [25, 26]. However, in our study, no significant differences in recurrence rates were found based on ethnicity or country of birth, suggesting that these factors did not play a major role in influencing recurrence within our patient cohort. Our study included 43 patients, with the following ethnic distribution: 28 Caucasians, 10 Africans, and 5 Hispanics. Despite the diverse ethnic composition of our sample, recurrence rates did not vary significantly across these groups (log-rank test = 1.1; *p* > 0.5), indicating that other factors may be more influential in determining recurrence risk in our population. In our study, however, we did not perform genotyping of *T. gondii* isolates; therefore, the infecting strain could not be directly assessed. For this reason, our analysis is based solely on ethnicity and country of birth, which may not perfectly reflect the geographic origin of the infecting parasite. Moreover, only 14% of patients showed positive IgM serology at first presentation, suggesting that most cases were less likely to represent primary infections acquired in Italy, although this possibility cannot be completely excluded. This limitation may partly explain why no differences in recurrence risk were observed, despite well-documented variability in strain virulence across different regions of the world.

#### Age

Regarding age, Garweg et al. [27] described in their study that patients under 21 years of age at the time of the first manifestation of ocular toxoplasmosis had a statistically significant higher risk of developing recurrences compared to older individuals. In line with this, Bosch-Driessen [5] and colleagues suspected that the risk of recurrence was low after the age of 45, although they were unable to confirm this hypothesis with analytical techniques. Holland et al. [28] also hypothesized in their study that recurrences were less common in older patients, presumably because most of them were infected at a younger age, and that the risk decreases with the time interval from the first episode. The average age of our sample was 45.7 years, with a range from 21 to 98 years. In our study, younger age was not found to be correlated with an increased risk of recurrence (log-rank test = 0.3; *p* > 0.5). On another note, De la Torre et al. [29] highlighted advanced age as a factor influencing the clinical severity and outcomes of ocular toxoplasmosis, attributing this to immunosenescence and the age-related decline in immune function; however, in our cohort, older age was not associated with an increased recurrence risk.

#### Primary Infection (IgM+)

Regarding the mode of infection transmission, 6 out of 43 patients (14%) had a postnatal infection confirmed by serological analysis (IgM+). However, in our study, the mode of transmission was not significantly associated with the recurrence probability (log-rank test = 1.2; *p* = 0.3).

#### Risk factors for reinfections

There have been hypotheses suggesting that reinfections could promote recurrences. It was previously believed that recurrences resulted from endogenous reactivation of dormant parasites within tissue cysts from previous infections. However, studies have reported reactivation of infections following reinfections [30, 31]. In our study, the analysis of possible risk factors for reinfection, such as cat ownership, jobs involving contact with soil, and dietary habits like the consumption of raw meat, did not show statistically significant correlations with recurrence.

#### Vitamin deficiency

Vitamin D deficiency, defined as serum levels below 20 ng/mL, has been associated with impaired immune system function and altered host defense mechanisms [32]. The association between vitamin deficiencies (particularly vitamin D) and the potential recurrence of toxoplasmosis did not show statistically significant results in our analysis (log-rank test = 0.2; *p* > 0.5). Two recent studies by Kashan et al. [33] and by Tayeb et al. [34] suggested a significant association between toxoplasmosis and vitamin D deficiency. However, it remains unclear from the studies whether *T. gondii* reduces vitamin D levels, thereby impairing the host’s immune defenses, or whether individuals with pre-existing lower vitamin D levels are more susceptible to contracting a Toxoplasma infection. In the study by Kashan et al. [33], the average vitamin D level was 9.9 ng/mL in the deficient group and 67.23 ng/mL in the group with normal levels.

#### Immunalteration factors

Our analysis also included the study of possible immunoalteration factors, associated with vaccines, and autoimmune diseases. Our sample did not include cases involving severe drug-induced immunosuppression or HIV-related immunodeficiency. The literature reports that immunocompromised patients have a higher risk of disease reactivation [10]. In our study, patients who had autoimmune diseases did not show a statistically significant correlation with an increased risk of recurrences. In line with the hypothesis of immune system stimulation, recent studies have documented the reactivation of ocular toxoplasmosis following COVID-19 infection and vaccination [35, 36]. However, in our study, no statistically significant association with vaccinations and COVID-19 infection was found.

#### Stress

Stress has long been suspected of playing a role in the etiology of various diseases, with several studies showing that it can exert an immunosuppressive effect, thus being harmful to health [37, 38]. It has been demonstrated that chronic stress alters immune responses [39, 40], shifting the cytokine balance from Th1 to Th2 responses [41], accelerating immunosenescence [42], and suppressing immunity by reducing the numbers and functions of protective immune cells, while increasing regulatory/suppressive T cells [43]. Our study demonstrated that stressful events can influence the reactivation of OT and the onset of new ocular recurrences. Regarding significant life events, such as mourning or university exams, reported by patients just preceeding the diagnosis or recurrences, these seem to affect the likelihood of recurrence: in the group that experienced significant changes, the number of recurrences was higher than expected, while in the group of patients that denied significant life changes, the number of recurrences was lower. Although the log-rank test (χ² = 3.3; *p* = 0.07) did not reach significance in this case, the data suggest modest evidence, indicating an association worth further investigation.

#### Sleeping habits/ duration of sleep

Sleep and stress are closely interconnected yet distinct factors in immune regulation. Regular sleep patterns have been associated with a more resilient stress response and better regulation of inflammatory processes [44]. It is possible that individuals with adequate sleep are less susceptible to the immunological effects of stress, or are better able to cope with stressful events, which in turn may reduce the risk of toxoplasmosis reactivation. Further research is needed to clarify whether sleep duration directly influences recurrence or whether it reflects broader lifestyle or psychosocial factors, including stress vulnerability. Among the analyzed factors, sleep duration was found to be statistically significant in influencing disease recurrence, with a lower risk for those sleeping between 6 and 8 h. A statistically significant difference (log-rank test = 6.8; *p* = 0.03) emerged for the sleep duration variable. Interestingly in our study, individuals who slept more than 8 h showed a higher probability of recurrence. Although regular sleep patterns generally support immune regulation, excessively long sleep duration has been associated in some studies with underlying health conditions, such as chronic low-grade inflammation [45], metabolic dysregulation, or increased fatigue related to unrecognized comorbidities. These factors could indirectly influence immune competence and could potentially contribute to increased susceptibility to reactivation of latent infections, including *T. gondii*. Another possibility is that prolonged sleep duration might reflect a compensatory response to chronic stress or poor sleep quality, rather than truly restorative sleep. In such cases, sleep quantity may not correspond to adequate sleep efficiency or circadian stability, both of which are important for balanced immune modulation. It is important to note that these interpretations are speculative and should be confirmed in future studies specifically designed to investigate the relationship between sleep duration and toxoplasmosis recurrence.

#### Localization

Lesions in Zone 1 which are also those most impactful from a visual perspective as they involve areas near the fovea and optic nerve, showed a lower recurrence risk than expected, while lesions in Zone 2, representing the mid-periphery and extreme peripheral retina, showed a higher recurrence risk. Although the log-rank test did not reach statistical significance (χ² = 3.5; *p* = 0.06), a noteworthy trend is observed and they indicate a potentially important direction for further studies.

#### Pregnancy

We also analyzed data on women who became pregnant after a diagnosis of OT and we found they had a recurrence risk three times higher than those who did not. In the pregnancy group, recurrences were numerically higher than expected, while, in the non-pregnancy group, they were lower. Although the log-rank test (χ² = 2.1; *p* = 0.1) did not reach significance, the estimate of the effect size (i.e. the hazard of recurrence in the pregnancy group compared with that in the non-pregnancy group) was as high as 3.1. The non significant result is mainly due to the small sample size (women were 15 in total), which limits the statistical power of the analysis. Our results align with a retrospective analysis by Kęcik et al. [46] on 213 women of childbearing age demonstrated a recurrence risk for toxoplasmic retinochoroiditis during pregnancy 7.4 times higher than during non-pregnancy periods (*p* < 0.001).

#### Drugs

**Fig. 2:**
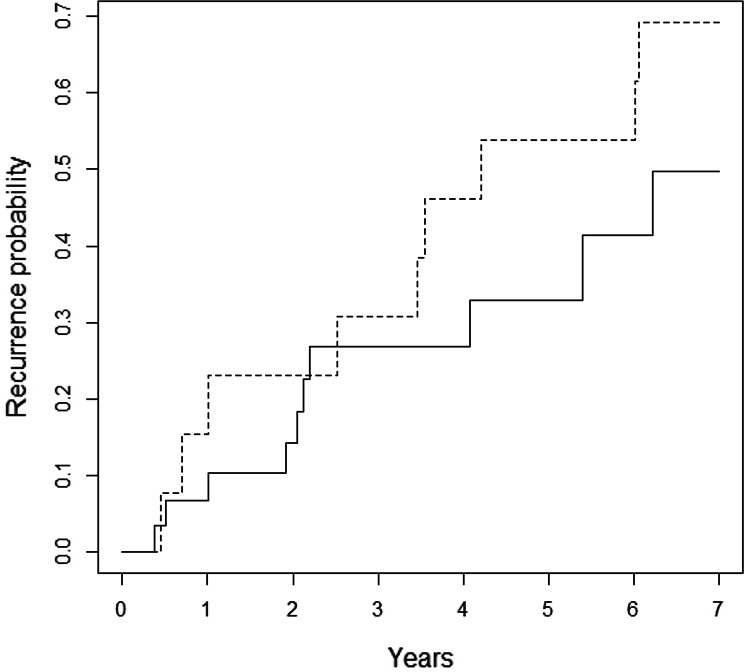
Recurrence probability according to two different drugs: trimethoprim-sulfamethoxazole (solid line) and pyrimethamine-sulfametopyrazine (dashed line). The x-axis indicates years of follow-up, and the y-axis indicates recurrence probability

No statistically significant difference (log-rank test = 1.7; *p* = 0.2) in recurrence probability was observed between the two drug classes (trimethoprim-sulfamethoxazole or pyrimethamine-sulfametopyrazine), even if the recurrence rate was numerically lower in the first group (0.8 recurrences per 10 person-years vs. 1.4 recurrences per 10 person-years), as well as the 6-year probability of recurrence: 0.413 vs. 0.538 (Fig. 2).

When the treatment effect on recurrence rates was evaluated separately in Caucasian and in non-Caucasian patients quite similar results were found: 0.9 recurrences per 10 person-years vs. 1.2 recurrences per 10 person-years (Caucasian) and 0.6 recurrences per 10 person-years vs. 3.2 recurrences per 10 person-years (non-Caucasian).

Similarly to our study, regarding the most effective drug for preventing recurrences, Rothova et al. [47] reported that no specific treatment regimen was superior to others in preventing recurrences.

#### Best Corrected Visual Acuity (BCVA) analysis

Finally, we compared the difference between initial BCVA values in logMAR at the first diagnosis and final values at the last follow-up using Welch’s test, considering the drug used (trimethoprim-sulfamethoxazole or pyrimethamine-sulfametopyrazine), lesion location, and the presence of recurrences. Regarding visual acuity at the first diagnosis based on lesion location, Group 1 (Location 1) had a mean logMAR of 0.51 ± 0.63, while Group 2 (Location 2) had a mean of 0.2217 ± 0.28. Although Location 2 appeared to be associated with better vision than Location 1, the difference was not statistically significant (*p* = 0.072). Initial visual acuity was modestly lower in Location 1, which, as expected, had a greater impact due to its proximity to central areas responsible for vision. The comparison of visual acuity before and after recurrence did not yield statistically significant results. Neither the number of recurrences nor lesion location appeared to significantly influence final visual acuity. However, a modest but non-significant trend suggested that patients treated with trimethoprim-sulfamethoxazole had better final visual acuity than those treated with pyrimethamine-sulfametopyrazine. This finding indicates that trimethoprim-sulfamethoxazole may help preserve visual acuity over time, although the underlying mechanism remains unclear.

## Conclusions

In our study, sleep duration emerged as a significant risk factor for recurrence of ocular toxoplasmosis, with patients sleeping between 6 and 8 h showing a lower risk of recurrence. Psychological factors, stress, and pregnancy post-diagnosis could appear to influence the recurrence risk, with women who became pregnant after diagnosis having a threefold higher risk of recurrence. Regarding the medications used, in our study no statistically significant differences were found between the two therapeutic regimens (trimethoprim-sulfamethoxazole and pyrimethamine-sulfametopyrazine) in terms of recurrence rates. Additionally, while previous studies reported a clustering of recurrences shortly after the initial episode and a decline over time, our data did not reflect such a pattern. In contrast, the recurrence rate in our cohort remained relatively stable during the first five years, with a cumulative incidence of 9% at one year and 58% at seven years, as illustrated in Fig. 1. This suggests that the risk of recurrence persists over time and does not necessarily diminish with longer follow-up, highlighting the importance of sustained long-term monitoring and potential prophylactic strategies. The results of our study could help identify individuals at higher risk of recurrence, and consequently, those who might benefit from secondary prophylaxis. Moreover, our analyses highlighted the period when the recurrence risk is highest, a crucial time when such treatment would be particularly important.

## Data Availability

The datasets used and/or analysed during the current study are available from the corresponding author on reasonable request.

## Abbreviations

OT: Ocular Toxoplasmosis
T. gondii: Toxoplasma gondii
BCVA: Best Corrected Visual Acuity
